# What Is Art Good For? The Socio-Epistemic Value of Art

**DOI:** 10.3389/fnhum.2017.00411

**Published:** 2017-08-28

**Authors:** Aleksandra Sherman, Clair Morrissey

**Affiliations:** ^1^Department of Cognitive Science, Occidental College Los Angeles, CA, United States; ^2^Department of Philosophy, Occidental College Los Angeles, CA, United States

**Keywords:** empirical aesthetics, neuroaesthetics, art appreciation, art as social practice, self-understanding, other-understanding

## Abstract

Scientists, humanists, and art lovers alike value art not just for its beauty, but also for its social and epistemic importance; that is, for its communicative nature, its capacity to increase one's self-knowledge and encourage personal growth, and its ability to challenge our schemas and preconceptions. However, empirical research tends to discount the importance of such social and epistemic outcomes of art engagement, instead focusing on individuals' preferences, judgments of beauty, pleasure, or other emotional appraisals as the primary outcomes of art appreciation. Here, we argue that a systematic neuroscientific study of art appreciation must move beyond understanding aesthetics alone, and toward investigating the social importance of art appreciation. We make our argument for such a shift in focus first, by situating art appreciation as an active social practice. We follow by reviewing the available psychological and cognitive neuroscientific evidence that art appreciation cultivates socio-epistemic skills such as self- and other-understanding, and discuss philosophical frameworks which suggest a more comprehensive empirical investigation. Finally, we argue that focusing on the socio-epistemic values of art engagement highlights the important role art plays in our lives. Empirical research on art appreciation can thus be used to show that engagement with art has specific social and personal value, the cultivation of which is important to us as individuals, and as communities.

“What art does is to coax us away from the mechanical and toward the miraculous. The so-called uselessness of art is a clue to its transforming power. Art is not part of the machine. Art asks us to think differently, see differently, hear differently, and ultimately to act differently, which is why art has moral force.”

— Jeanette Winterson (Winterson, 2006)

## Introduction

Traditionally, discussion of the nature of the arts and their role in our daily lives and communities lay within the purviews of criticism, art history, and philosophy. Within the last century, there has been a growing interest by psychologists and more recently, neuroscientists, to scientifically investigate art experiences and appreciation. Broadly, questions central to this investigation include:

- What happens when we experience a work of art? Specifically, what are the perceptual, emotional, and cognitive processes mediating our responses to art?
- Can one account for variations in taste? And if so, how does one's psychology and biology contribute to those preferences?
- What is common about the experiences one has across different forms of art? What is distinct?
- Are our responses to art universal or culturally and historically situated?
- Are art experiences pleasurable and how is the response distinct from other pleasurable experiences?

To scientifically investigate these questions, psychologists often ask viewers to rate the aesthetic appeal of an artwork, to rate their preferences for it compared to other artworks, and to indicate their emotional responses to various works. Typical questions might include: how much do you like the artwork; how aesthetically pleasing is the artwork; and how emotionally moving is the artwork? Researchers might then analyze the extent to which ratings reflect the formal features of that artwork—e.g., how balanced the composition is, how prototypical the depictions are, or perhaps how much the statistical structure within the image parallels natural scene statistics. As such, psychologists have identified a variety of formal features that seem to influence aesthetic and preference scores, including symmetry, color, contrast, aspect ratio, prototypicality, natural scene statistics, and complexity (e.g., Berlyne, 1971; McManus, 1980; Taylor et al., 1999; Shortess et al., 2000; Graham and Field, 2008; Schloss and Palmer, 2011). Similar questions have been explored in other domains of art including music and literature (e.g., Rentfrow and Gosling, 2003; Koopman and Hakemulder, 2015). Furthermore, many researchers have demonstrated that individual differences, be they stable or transient, can influence preferences and judgments. For instance, culture and experience (e.g., Reber et al., 2004; Bullot and Reber, 2013), expertise and knowledge (e.g., Winston and Cupchik, 1992; Silvia, 2006) and current emotional state (e.g., Eskine et al., 2012) shape judgments. Additionally, individual differences in perceptual capacities, such as visual-object working memory (VOWM) are associated with preferences for formal features such as visual complexity within visual artworks (Sherman et al., 2015). These findings aim to illustrate the importance of accounting for the between and within subject variability in preferences, emotional responses, or beauty judgments.

A complementary approach, neuroaesthetics, is concerned with investigating the neurobiological substrates of aesthetic experience. For example, studies employing fMRI often task participants with making aesthetic or emotion-related judgments, and have demonstrated that art appreciation activates a distributed network in the brain subserving three core neural systems: sensory-motor, emotion-valuation, and meaning-knowledge. Important regions linked to aesthetic evaluation and preference for art include areas related to domain-specific processing such as the visual system for visual art (e.g., the lingual gyrus, middle occipital lobe), memory recognition (e.g., fusiform gyrus, parahippocampal gyrus), higher-order conceptual integration (e.g., anterior temporal lobe), emotion and reward (e.g., the anterior insula, caudate/striatum), valuation (e.g., anterior and ventromedial prefrontal cortices), and more recently metacognition (e.g., structures within the default mode network such as posterior cingulate cortex) (for reviews and meta-analyses, see Di Dio and Gallese, 2009; Brown et al., 2011; Chatterjee and Vartanian, 2014; Vartanian and Skov, 2014).

Notably, although the aesthetic sciences broadly concern themselves with explaining art appreciation^1^, what can be gleaned from the above findings is that they have, up to this point, primarily investigated experiences of the aesthetic. That is, scientists have privileged investigating individual judgments of beauty or preference, many times ignoring socially-relevant outcomes of art appreciation or the social context of art creation and art appreciation. This is the the case within both the psychological and neuroaesthetics literatures. For example, neuroaesthetics research typically uses art (paintings, music, poetry, dance performance) as a stimulus to determine the neural mechanisms associated with preference, beauty, sublimity, and pleasure-based responses (e.g., Blood and Zatorre, 2001; Kawabata and Zeki, 2004; Vartanian and Goel, 2004; Jacobsen et al., 2006; Ishizu and Zeki, 2011; Lacey et al., 2011; Brattico, 2015).

Empirically investigating art appreciation in this way, however, risks conflating the arts with aesthetics. That is, it risks reducing the study of the nature of the arts to their ability to cause a particular feeling of disinterested joy or pleasure in a beholder. This reduction is reflected in (i) the way neuroaesthetics frames and understands art—namely, as an object that one contemplates and experiences in a disinterested manner, (ii) in the focus researchers place on measuring judgments related to beauty, liking, and pleasure as primary “outcomes” of the art experience, and (iii) in the contexts in which aesthetic experience is studied, often in labs on computers, removed from social and historical contexts, and in the visual arts, over short viewing times rarely exceeding 15 seconds.

The prevailing use of these measures and contexts implies that what defines an art experience is the pleasure caused by interacting with something aesthetically pleasing, and that the primary scientific task is describing the perceptual and emotional processes related to, or which constitute, a moment of liking or joy. Such a reduction limits the range of human experiences and capacities identified as appropriate objects of scientific investigation in this field. Moreover, “able to cause aesthetic experience” is a philosophically dubious conception of the nature of the arts, and can be particularly problematic in cases where “beauty” or “disinterested pleasure” is not a productive theoretical framework for evaluation of an artwork, as in some modern and contemporary art forms (e.g., see Carroll, 2012 for review). Similar methodological critiques have been presented within music as well as other domains of art (e.g., Sloboda et al., 2001; Brown and Dissanayake, 2009). For instance, within the domain of music, much of the research investigates individuals' cognitive and emotional responses to passively listening to a musical piece (as well and the perceptual features that prompt such a response) discounting the social functions of the work.

Frameworks from the history of philosophical aesthetics and contemporary methodological discussion within empirical aesthetics can be particularly instructive for psychologists and neuroscientists interested in investigating the arts. As indicated above, philosophical attempts to define the nature of art by appeal to the kind of experience often studied by aesthetic science have been criticized for failing to fully capture or appreciate the social, cultural, or historical situatedness of the art-object or the person whose experience is being studied. Some empirical contextualist theories take a similar stance, recommending scientific investigations that go beyond the “basic exposure” mode of art appreciation, noting that the kind of knowledge one would gain from perceptual exploration removed from historical understanding is “shallow at best” (Bullot and Reber, 2013). Rather, psychology must embrace an enriched understanding of art appreciation by investigating how, for example, an individual causally reasons about the observable features and attributions of an artwork, “mindreads” or attempts to cognitively model the artist and her intentions, experiences discovery or understanding-based emotions, and generates theories about the relevant content, form, and function of the artwork (Bullot and Reber, 2013).

Relatedly, we suggest that the current scientific research on art appreciation discounts what many would consider the very essence of art: its communicative nature, its capacity to encourage personal growth, its ability to reveal deep aspects of the human condition, to challenge preconceptions, to help us reconceptualize a question we are grappling with, and to provide clarity on ambiguous concepts or ideas. A host of philosophical, art-historical, and critical theories of the nature of the arts, art appreciation, and artistic creative practice suggest a more general theoretical shift away from the project of empirically studying art-objects by focusing on individuals' phenomenological experiences, and toward one which recognizes that individual psychological experiences or habits are shaped by engaging with the arts as part of our communities and social fabrics (e.g., see Carroll, 2012 for review). For instance, some philosophers and scientists alike have claimed that the arts, broadly conceived, have moral value, suggesting that engaging with art can be potentially transformative, for it encourages us to consider the welfare and good of other people, enhancing both our moral compass and self-knowledge (e.g., Nussbaum, 1990; Koopman and Hakemulder, 2015).

Our primary goal here is to argue that a systematic scientific study of art appreciation must explain the potentially broad-ranging and diverse social outputs of arts engagement, and thus, must go beyond measurements of aesthetic pleasure or liking. We advocate for the need to embrace an expanded empirical research program characterized by reframing the arts as *socio-epistemically valuable*—that is, specifically useful for gaining knowledge and insights about oneself and society. Importantly, we suggest that an empirical research program that recognizes the arts as social practices (which we expand in Section Arts-Appreciation as Socio-epistemically Valuable) can potentially unify prior research and more clearly specify the types of investigations needed to achieve a fuller understanding of art appreciation.

For instance, information-processing accounts of art appreciation aim to understand the relationship between inputs (e.g., formal features, transient individual differences like emotional or mood states, and more sustained individual differences in personality, culture, historical contexts, or expertise), processing mechanisms unfolding related to the art experience (e.g., the psychological and neurobiological substrates of perceptual, cognitive, and emotional processes, or disruptions to one's self-schema), and outputs (e.g., appraisals/judgments of liking, epiphany/transcendence, self/other-understanding; well-being). Fitting to our art-as-social-practice view, we suggest that researchers might begin to investigate the information-processing system through the lens of socially-related outputs, such as self and other understanding, rather than through the lens of aesthetic outcomes of art (see Table 1). That is, how do brain structures like the default mode network, which is recruited during art appreciation, contribute to socio-epistemic outcomes of art appreciation like self-understanding? This focus may reveal the need to develop experimental approaches better suited to evaluating the nature of the arts which recognize how creative practices and appreciation are cultivated socially, over long periods of time, and sustained both at the community and the personal level.

**Table 1:** Factors influencing art appreciation.

**Inputs**	**Processing mechanisms**	**Outcomes**
**Self-related**	**Implicit**	**Immediate**
States (e.g., mood, affect, attention) Traits (e.g., self-concept, social schemas, personality, cognitive and perceptual capacities) Prior experience (e.g., domain specific expertise, memory, tastes, interests, culture)	Perceptual analysis Memory integration Embodied simulation Emotional resonance Initial classification	Emotional appraisal (e.g., negative, positive, mixed emotions) Aesthetic decision/evaluation (e.g., preference, pleasure, like/want, good/bad) Bodily/physiological response (e.g., chills, tears, arousal) Insight and/or epiphany
**Object-centered**	**Explicit**	**Longitudinal**
Formal properties (e.g., symmetry, statistical profile, harmony, dynamism, style) Meaning-related content **Environmental context** Sensory information (e.g., noise, temperature, lighting)	Directed attention Evaluative criteria (e.g., relevance, intentionality, style, content) Metacognitive awareness (i.e., self-monitoring) Self-reflection Meaning-making	Social knowledge Self-understanding (e.g., belief/schema revision) Other-understanding (e.g., developing empathy, perspective-taking, “practice” mentalizing) Well-being/flourishing/health Perceptual skills (e.g., visual discrimination) Cognitive skills (e.g., creativity)

*An information processing account of art appreciation denoting self and other referential processing as well as the immediate and longitudinal socio-epistemic outcomes. Note that this table lists factors and processing mechanisms relevant to art appreciation but does not highlight the temporality or connectivity between the factors. For a review of models that differ on these dimensions, see Pelowski et al. (2016)*.

Below, we start by framing the arts as social practices that are embodied, enactive, and communicative. Although our art as social practice organization is not in contrast to information processing accounts, it importantly allows us to focus empirical evaluations on the cluster of skills that are developed through art appreciation. Among these skills, we focus specifically on those we refer to as socio-epistemic, and demonstrate that self- and other-understanding are both socially relevant and meaningfully cultivated through sustained art engagement.

## Arts-appreciation as socio-epistemically valuable

We begin by situating arts engagement, and specifically art appreciation, as a communicative, dialogic, dynamic, and transformative practice rather than as passive contemplation of beautiful, pleasurable, or otherwise aesthetically interesting objects. We argue that an “art as social practice” framing like this raises more relevant, interesting, and psychologically rich questions about the arts than does the traditional framing of art appreciation as reducible to aesthetic experience.

### The arts as social practices

In *Art Rethought: The Social Practices of Art* (2015), Wolterstorff argues that we should adopt MacIntyre's account of social practices as a framework for understanding the nature of the arts (Wolterstorff, 2015). MacIntyre (1984) defines social practices as:

…coherent and complex form[s] of socially established cooperative human activity through which goods internal to that form of activity are realized in the course of trying to achieve those standards of excellence which are appropriate to, and partially definitive of, that form of activity, with the result that human powers to achieve excellence, and human conceptions of the ends and goods involved, are systematically extended (p. 187).

As forms of human cooperative activity, they exist within social groups, both large and small, and persist through time. Consider, for example, the social practice of portraiture, a genre of painting which depicts a human subject, often in which the face is the main theme. This genre has existed historically across many, varied communities, and the genre develops and is shaped by the cultural, economic, and moral commitments of various social groups, in addition to the artistic styles and technological developments within these communities. “Painting a portrait” is done with respect to norms, standards, and expectations of the genre that are, in an important sense, public. Moreover, these norms and standards constitute criteria for having created an excellent portrait. That is, we can individually and collectively deliberate and debate about whether some particular artwork is a portrait, or is a *good* portrait. Furthermore, accomplishments such as ‘mastering the ability to depict a complex emotional expression in a two-dimensional medium’ (Leonardo DaVinci's *Mona Lisa*), or ‘successfully communicating the cruelty of poverty and dignity of poor people by rendering sympathetically and beautifully the humanity of someone who is poor’ (e.g., Dorothea Lange's, *Migrant Mother*), are goods that can only be achieved through the practice of portraiture. Finally, the genre, itself, develops throughout time, within different communities. There are innovations in portraiture with respect to artistic style and with respect to technology. Consider, for example, how Henri Matisse's *Green Stripe (Portrait of Madame Matisse)* both radically departs from and conforms to the norms of the practice, or how the invention of photography changes and informs the meaning of “creating a portrait.” Matisse's innovation and the development and use of photography for artistically depicting human faces, both enrich our understanding of the aims of art and the possibilities of human experience.

By following this emphasis on the arts as practices, we mean to shift attention to art creation and art appreciation as activities “we do,” from the conception of art appreciation as passive reception of perceptual information from art-objects. In doing so, we do not commit ourselves to any particular theory or definition of art, be it the institutional view (Danto, 1964; Dickie, 1974), which holds that artworks are artifacts that have been identified as such by persons appropriately situated with respect to “the artworld,”^2^ or the historical (Levinson, 1979) or narrative views (Carroll, 1988), which hold that artworks can be identified by relationships to existing artworks. Instead, we follow these traditions, and others in anthropology and sociology (e.g., Becker, 1982; Dissanayake, 1990; Gell, 1998; Harrington, 2004), in their recognition that both arts appreciation and art creation, whatever they may be, are culturally situated within human communities^3^. We contend that this very foundational and basic recognition is largely absent or significantly downplayed in current empirical work, and it is this sense of social—longstanding practices, embedded in the fabric and life of communities—that is foundational to our proposed framework.

### The arts and socio-epistemic skills

One model for how to understand art appreciation as active engagement in a practice can be found in Kieran (2012). There, he argues that art appreciation is an intrinsically valuable skill that allows one to cultivate “excellences of character,” because practiced arts engagement allows one to better imagine and critically examine not only aesthetic qualities of artworks, but also “artistic originality, emotional expression, insight and moral understanding.” (p. 23) This notion of skill has a few different features that matter a great deal to an expanded empirical research program: (1) art appreciation is learned through sustained practice, suggesting its intrinsic relationship to the culture and community, or, at least, to other people; (2) is a capacity that is developed over non-trivial lengths of time; and (3) may be relevant to other domains, as skills can be transferable.

Drawing from other philosophical literature on art appreciation, we see a focus on what we refer to as socio-epistemic skills. Included in this category may be capacities like good judgment, richer sensitivity to detail, or, following Hume, “delicacy of imagination, good sense, comparative experience, and freedom from prejudice” (Kieran, 2012, p. 23). What makes these skills social is their relationship to one's ability to better understand oneself and other people, and to potentially revise one's own moral, political, or social commitments^4^. Although the mechanism for enhanced understanding of self and others is not fully theorized in the philosophical literature, it is often taken to be developing a kind of sensitivity to detail, context, or nuance (e.g., Murdoch, 1970; Nussbaum, 1990; Carroll, 1998).

Empirical research complements the philosophical framework above by helping us understand the mechanisms that underwrite the particular socio-epistemic skills of other-understanding and self-understanding^5^. We choose to highlight self-understanding and other-understanding because they align well with what many think of art appreciation as doing: helping them see others and the world from a different point of view, altering their perspectives, and helping them to understand more about themselves (e.g., what moves them, or what makes them uncomfortable). At the same time, we do not mean to commit to any specific or direct causal pathways between cognitive processes, art appreciation, and other- or self-understanding. Rather, we mean to identify this as an open area of much needed investigation.

Before turning directly to this discussion, we also note that embracing this theoretical shift toward understanding the arts as social practices would allow us to explain how art appreciation is partially constitutive of living a flourishing human life. A longstanding empirical program has been to connect the arts (both appreciation and creation) to happiness, well-being, or flourishing. For instance, Cuypers et al. (2012) demonstrate through a large-scale population study that both art appreciation and art creation are associated with increased well-being (as measured by perceived health, life satisfaction, and anxiety and depression scores). Philosophical conceptions of *eudaimonia* contend that a flourishing human life centrally involves, at least, the use of skills or excellences of character the development of which are intrinsically rewarding, and the exercise of which are, thereby, pleasurable. Thus the shift we are recommending does not discount previous research, but rather, locates and explains the liking, preference, and pleasure responses to art-objects as well as the experience of being moved, as important aspects of the skill-based conception of art appreciation. This also allows us to strengthen arguments for the value of the arts that does not embrace crass instrumentalism, but rather, is capable of explaining the central role of the arts in human life (Kieran, 2012). Moreover, regardless of whether one is committed to the broader eudaimonistic theory of well-being, or the claim that the development of human excellences and skills is central to that flourishing, those who hold that art appreciation is capable of developing the capacities and related skills of other-understanding and self-understanding are making *empirical* claims that empirical aesthetics can evaluate. To that end, a complete model of aesthetic appreciation will also need to contend with these claims and find a place for these socio-epistemic “outputs” in their models.

In the sections that follow, we use philosophical discussions to frame and suggest two lines of empirical inquiry within this theoretical orientation of the arts as social practices. The first, self-understanding, discussion of which is nascent in both the psychological and philosophical literatures, asks whether and how art appreciation as a practice can lead to a richer understanding and appreciation of one's own moral values, commitments, and conception of who and what one is. The second, other-understanding, more fully developed in both literatures, asks whether and how art appreciation as a practice can lead to a better understanding of the emotional and cognitive states of others, and the potential moral and social value of such an understanding. We conclude with a discussion of how such a research program may be envisioned and developed moving forward.

## Art engagement as a path to self-understanding

As discussed above, in this section we attempt to lay a foundation for a line of inquiry into how self-understanding may be enhanced by engaging in practices of art appreciation, as part of our suggestion that conceptualizing the arts as social practices would be an appropriate and fruitful framework for psychologists and neuroscientists to embrace.

### Philosophical conceptions of the relationship between art appreciation and self-understanding

In philosophy, the term “self-knowledge” often refers to knowledge of one's own mental states—that is, knowledge of our own beliefs, thoughts, or sensations. In contrast, “knowledge of the self” can refer to knowledge or understanding of one's “self” and its nature. Following Gertler (2015), we may include under this heading four different debates about our understanding of ourselves, as selves: the nature of self-identification (i.e., one's ability to distinguish one's self from others); whether self-awareness is a mechanism for grasping the nature of the self; whether self-awareness is a means to grasping one's personal identity over time; and, whether and what sort of self-understanding is necessary for rational or moral agency.

Insofar as engagement with the arts is able to enhance some notion of self-understanding, it fits most comfortably within this final debate: the sort of self-understanding necessary for rational or moral agency. Martin (1985), providing one way of enriching this “necessary for agency” conception, claims that self-understanding is an *achievement*. He explains that developing a “justifiable and meaningful perspective on our lives” often calls for “appropriate adjustments in attitude, emotion and conduct,” and realizing these things is something that we work for, or that we strive to accomplish. (p. 2) Relevant to this kind of self-understanding is what we may refer to as “self-identity”—“individuals' subjective senses of who they are—their own self-images” (Martin, 1985, p. 5). Further, we may consider the heart of self-identity as a set of commitments or values—be they intellectual, artistic, moral, or religious—that organize individuals' behavior, attitudes, and beliefs. Someone who has proper self-understanding not only recognizes and affirms her central commitments and values, but also acts and feels according to these commitments and values. In this way self-understanding is a socio-epistemic skill because one's ability to recognize and act on her central values (e.g., feel and act compassionately) concerns a *social* ability. The content of the values or commitments substantially refer to other people, institutions, histories, and communities, and the attitudes and behaviors indicated are learned and exhibited within communities and relationships.

Philosophers who defend the view that art appreciation is a form of moral understanding can inform our conception of how art appreciation may enhance self-identity and self-understanding. A particularly influential view is Noël Carroll's clarificationism (Carroll, 1988). Unlike the sciences, which allow individuals to acquire new propositional knowledge, Carroll argues art appreciation is capable of deepening our existing knowledge, something he refers to as “understanding.” Carroll suggests that the narrative arts, in particular, encourage us to apply our moral knowledge and emotions to a specific case, which aids in the development of our capacity to manipulate, refine, or clarify what we know, and to then intelligibly apply that knowledge. Carroll uses the example of *Crime and Punishment* to explain this point. It would be absurd to claim that the reader learns the truth of the proposition “murder is wrong” from her reading of the novel. In fact, it may be that a reader would already need to have this bit of propositional knowledge in order to make sense of the novel in the first place. Yet, engagement with the novel can be a source of moral understanding and self-development. Engagement may help give shape to, clarify, or deepen one's understanding of the horror of killing, and of the nature or importance of guilt, redemption, and moral character. Moreover, insofar as these moral beliefs and values are part of the central commitments and values that constitute your self-identity, engagement with the novel can help you know yourself better.

That art is a context for deepening understanding rather than gaining propositional knowledge is also taken up by Lopes (2005). There he argues that the kind of seeing (“seeing-in”) cultivated by practiced visual art engagement enriches moral sensibility by enriching the suite of intellectual resources that make the viewer reliable at discriminating morally relevant features of situations. (p. 180) Part of the moral sensibility Lopes describes includes what he refers to as a repertoire of moral concepts (e.g., solidarity, grief, violation). Some visual art, though not all according to Lopes, can be used to deepen and understand those concepts. In this way, some visual art can communicate moral ideas in new or challenging or poignant ways that cause one to revise an important or closely held moral value, and thus, can be important to developing one's self-understanding.

Although the philosophical discussion of self-understanding or transformation through engagement with the arts primarily concerns moral or social knowledge, we see no reason to believe it must be limited to these contexts. The focus on moral knowledge in the philosophical literature may be occasioned by the felt need to distinguish the arts from the sciences as a means of knowing, as the latter tend not to have this moral or social focus^6^. However, we may think of the arts as a path to non-moral self-understanding as well, or, as above, as about non-moral yet central commitments and understandings important to our self-identity. For example, the works displayed during the 2013–2014 Los Angeles County Museum of Art retrospective of the work of *Light and Space* artist James Turrell, were described by many (critics and lay people alike) as *transformative*. The immersive light environments cause one's own perception to become the object of reflection, and led many to a deeper understanding of themselves and their relationship to the external world, deepening their conception of themselves as embodied beings whose access to the world is *mediated by a visual perceptual faculty* with particular features, limitations, and abilities, and of light, itself, as a physical substance. This fact (that perception is mediated by light) is not one that people learn from this exhibit; people learn that in middle school science classes. But being confronted with artistic works that exploit and make manifest this fact nevertheless affords viewers an understanding of the significance of this fact.

### Enhanced self-understanding through art appreciation: empirical evidence

As in the philosophical literature, there also seems to be limited work in the psychological literature focused on the importance of art engagement in cultivating self-understanding, although research on self-reflection may speak to the psychological mechanisms that make possible the socially-relevant conception of self-identity as described above. Following Koopman and Hakemulder (2015), self-reflection refers to “thoughts and insights on oneself, often in relation to others and/or to society” (p. 82). This type of introspection often relates to one's emotions (e.g., monitoring current states and/or comparing those states to prior states), memories, values, and beliefs, and is associated with positive consequences (e.g., better mental health, well-being, increased capacity for self-regulation).

The literary arts are a domain in which self-reflection has received more comprehensive attention. Koopman and Hakemulder review evidence suggesting that self-reflection is elicited when one reads literary texts characterized by unconventional syntax or semantic features. Specifically, they review empirical work showing that self-reflection occurs in scenarios in which “(i) [reader's] previous personal experiences are evoked by descriptions of characters, places and events, (ii) [in which] readers experience emotional responses to the characters, and (iii) [in which] readers perceive the text itself, the artifact, as striking” (p. 95). Self-reflection elicited through reading in these contexts is likely to relate to one's self-understanding and identity both in moral and non-moral contexts. Similarly, some members of the medical community have embraced the idea that the literary and narrative arts facilitate self-reflection. Brady et al. (2002) posit that practicing self-reflection outside of a clinical context, and particularly through art appreciation, could lead to better doctor-patient relationships and, thereby, better patient outcomes.

With respect to visual art, research in neuroaesthetics has also suggested that when engaging with artworks that are emotionally moving and potentially transformative, individuals may have an inward, self-reflective focus. Here, being moved refers to “intensely felt responses [such as tears or chills] to scenarios that have a particularly strong bearing on attachment-related issues—and hence on prosocial bonding tendencies, norms, and ideals—ranging from the innermost circle of one's personal life … to higher-order entities of social life (one's country, social and religious communities)” (Menninghaus et al., 2015, p. 8; see also Hanich et al., 2014; Wassiliwizky et al., 2015, 2017a). Recent work by Wassiliwizky et al. (2017b) suggests, for example, that poetry containing a socio-cognitive component (e.g., prose addressing other people or personifying nature) is particularly moving, leading to chills and a response in brain areas involved in self-reflection (e.g., precuneus). When an artwork moves a beholder, she likely experiences an intense emotional response as well as explicitly reflects on her experience, potentially exercising self-understanding (as well as other-understanding, which we expand on in the next section). In this way, understanding the experience of being moved (rather than just focusing on aesthetic evaluation) indicates a promising avenue of research for neuroaesthetics to develop in line with our recommendation to adopt a social practice model.

Indeed, Vessel et al. (2012, 2013) have demonstrated that during intensely moving aesthetic experiences, the default mode network—a network of brain areas including the precuneus, medial frontal cortex, inferior parietal cortex, and medial temporal cortex known to be involved in self contemplation, self reflection, and self-referential thought—is recruited. In Vessel et al.'s (2012, 2013) studies, participants were tasked with attending to a set of visual artworks and judging how moving each one was while their brain activity was recorded in a scanner. Their finding that DMN activity was higher for artworks rated as highly moving relative to those rated lower on the scale may be interpreted as an inward, self-reflective focus that co-occurs with or is prompted by being emotionally moved. Additionally, this finding is consistent with research demonstrating that the DMN is recruited during other self-referential types of tasks involving self-identity (namely, making judgments about yourself or close others), moral decision-making, and theory of mind attributions (Northoff and Bermpohl, 2004; Northoff et al., 2006).

Psychologists have also described models that center the idea that art appreciation recruits metacognitive processes and promotes self-reflection and transformation. For example, Pelowski and Akiba (2011) (see also Pelowski, 2015; Pelowski et al., 2017) argue that influential empirical studies of aesthetic experience focusing on understanding the processes which lead to cognitive mastery of an artwork along with perceptual pleasure are “often divorced from a viewer's personal beliefs and identity” and “preclude the possibility for art to [truly] mark and transform lives” (p. 81) namely because they do not directly address discrepant experiences during an art encounter. According to Pelowski and Akiba's account, the self-reflective processing that occurs when a beholder's expectations have been violated (e.g., confusion about meaning) marks the beginning of a meta-cognitive re-assessment of an artwork, eventually leading to self-schema transformation. Similarly, Lasher et al. (1983) argue that the arts are central for mental and emotional growth because they offer opportunities for representational conflicts that, when resolved (in their case, often unconsciously) provide a way to restructure and unify initial mental representations. The process of defamiliarization, “becoming unsettled,” and self-reflecting, then may be crucial to deepening self-understanding.

In a more recent paper, Pelowski (2015) offered an empirical approach to studying art experiences as they relate to self-transformation and understanding. Specifically, Pelowski suggests that feeling like (or actually) crying during an art experience is a physical indicator of self-reflection, shifted perspectives, and self/schema changes. As a first foray into testing his model, Pelowski conducted a series of exploratory studies at several museums collecting both physiological data and self-reports from museum-goers. He demonstrated that feeling like crying while viewing art is correlated to increased self-awareness, feelings of epiphany and insight, as well as to mixed emotions corresponding to being moved. Although his empirical findings are specific to the visual arts, his model broadly appeals to all arts, as tears or chills responses are pervasive across all arts domains (Pelowski, 2015). Pelowski's approach is particularly instructive as it offers a means to frame socio-epistemic skills such as self-understanding within information-processing accounts, arguing for the importance of empirically investigating how each processing stage corresponds to self-related outcomes.

Importantly, these ideas are markedly different from the more typical information-processing accounts of aesthetic experience (e.g., Leder et al., 2004 or Chatterjee, 2004), which focus more on successful assessment of an artwork's formal information (perceptual and cognitive mastery) in the service of emotional appraisals. This traditional approach de-centers the importance of self-reflection or cognitive growth as an outcome or aspect of art appreciation. In contrast, the paradigm we suggest (which parallels Pelowski's) posits that although detached, the contemplative pleasure, which may be an outcome of art appreciation, is not valuable merely for its own sake, but also instrumentally valuable for deepening one's self-understanding.

Although the reviewed studies are not direct evidence that self-understanding is developed by art appreciation, they suggest, at least, that self-reflection, a process relevant to cultivating self-understanding, is prompted by moving art experiences. More research will be needed to understand the extent to which and how neural mechanisms correlated to self-referential processing are recruited during art appreciation. Candidate regions for investigation are those within the cortical midline structures including the orbitomedial prefrontal cortex (OMPFC) implicated in the *continuous representation* of self-referential stimuli and in processing emotional stimuli independent of sensory modality, the dorsomedial prefrontal cortex (DMPFC) implicated in *evaluation* of self-referential stimuli, the anterior cingulate cortex (ACC) implicated in *monitoring* of self-referential information, and the posterior cingulate cortex (PCC) and adjacent precuneus thought to be involved in self-reflection and the integration of self-related representations (e.g., Northoff and Bermpohl, 2004). The partially overlapping default mode network as described above will also be critical to evaluate in the context of art appreciation.

## Art engagement as a path to understanding others

Turning away from self-understanding, in this section we lay a foundation for a line of inquiry into how other-understanding may be enhanced by engaging in practices of art appreciation. Though here we highlight self- and other-understanding as separate socio-epistemic skills, we also point to the importance of investigating these “outcomes” as highly related. As before, the aim of this section is to build our suggestion that conceptualizing the arts as social practices would be an appropriate and fruitful framework for psychologists to embrace.

### Philosophical conceptions of the relationship between art appreciation and other-understanding

Philosophers of art commonly contend that art appreciation enables us to understand others better by encouraging us to take on their viewpoints, to metaphorically take a walk in their shoes, to feel their pain. Through art appreciation we can understand ourselves as connected to one another, by recognizing others' emotions, actions, and perceptions as fundamentally similar to our own, or, more dramatically, by *feeling* others' emotions. For instance, in Cohen's (1993) discussion of his ambivalence toward ontological questions about the nature of art and the distinction between high and low art, he describes a memorial service in which his friend's favorite musical selections were played. Reflecting on the meaningfulness and appropriateness of this practice of playing music that someone cared for at their funeral, Cohen writes:

My friend has died and is not present. I listen to music I know he cared for. It is a fact about my friend that he cared for this music, perhaps even a constitutive fact about his sensibility: it partially defines who and what he was. It is, thus, an entrance into that sensibility. I sit listening, not merely thinking that this music meant something to my friend, but bending my imagination to the task of reaching and comprehending an aspect of my friend which responded to this music, that is, feeling what it was to be my friend (p. 154).

Here, Cohen understands artistic appreciation not only as (appropriately) playing a central role in an important social ritual of mourning, but also, or perhaps because it is one way of being in community with someone else. In this case, the mind, sensibility, or self of the person who is no longer present is accessible through attending closely to the music he loved. Similarly, Joseph Conrad characterizes the emotional sharing involved in artistic activity as:

the subtle but invincible conviction of solidarity that knits together the loneliness of innumerable hearts; to the solidarity in dreams, in joy, in sorrow, in aspiration, in illusions, in hope, in fear, which binds men to each other, which binds together all humanity—the dead to the living and the living to the unborn (cited in Goldie, 2008, p. 192).

This notion, that the arts are an arena for interaction and potential emotional sharing between artists, beholders, and other past, present, and future beholders has an important history stretching back to at least Tolstoy (1899), if not to Aristotle.

The kind of interaction or connection art facilitates has been thought to lead to a fuller and morally important understanding of others and oneself. Kieran (1996) develops a notion of “imaginative understanding,” a skill promoted by the arts, as striving to “appreciate what the appropriate way of looking at and acting in the world is…typically…the appropriate way to feel for, to regard, and to respond to others” (p. 341). In this way, art appreciation, by promoting imaginative understanding, facilitates good moral judgment by enhancing our moral perception and sensibilities, especially with respect to the lived experiences of other people^7^.

Developing a similar line of thought, some scholars have suggested that reading literary fiction creates aesthetic distance, which “allow[s] [readers] to experiment more freely with taking the position of a character different from themselves, also in moral respects” (Koopman and Hakemulder, 2015, p. 92). That is, the dynamic process occurring during art appreciation is a form of socio-cognitive and emotional training, granting viewers the “time and privacy to learn to deal more strategically with” real life scenarios in a safe, “distant” space (this idea has been discussed by Oatley, 1999, 2016; Robinson, 2005; de Botton and Armstrong, 2013; Koopman and Hakemulder, 2015; Menninghaus et al., 2017). Despite this “distance” or, perhaps because of it, one can become deeply invested in fictional characters, emotionally engaging with them, and generating cognitive models of character's minds, just as one does in real social scenarios^8^.

That arts appreciation can deepen one's moral landscape by cultivating other-understanding is an empirical claim with potentially far-reaching consequences^9^. This idea has served as a theoretical foundation for arts-based therapies aimed at developing, for example, autistic children's social skills and theories of mind (see: arttherapy.org). Perhaps most robustly, as we briefly mentioned, in recent decades medicine has increasingly turned to the arts to help students and professionals cultivate proper self- and other-regarding dispositions (Shapiro et al., 2009). For example, Columbia University's Masters of Science curriculum in Narrative Medicine uses the arts and humanities to “imbue patient care and professional education with the skills and values of narrative understanding” (see: http://ce.columbia.edu/narrative-medicine). Some have suggested that arts-based interventions help physicians become more empathic and culturally-sensitive, which then leads to better patient health outcomes (e.g., Novack et al., 1997, pp. 502–509), whereas others have focused on the importance of reflection and imagination for developing insight, emotional understanding of patients, or other valuable “patterns of knowing” (e.g., Berragan, 1998; Rodenhauser et al., 2004; Averill and Clements, 2007).

These theoretical applications demonstrate the importance of reviewing the available empirical evidence that aligns with an argument that art appreciation cultivates other-understanding, the importance of understanding the psychological mechanisms underlying other understanding, as well as the importance of establishing norms for empirically investigating more fully the socio-epistemic outcomes and values of art appreciation.

### Enhanced other-understanding through art appreciation: empirical evidence

Psychological research suggests that there are (at least) two related ways we can come to understand other people and their experiences: (i) cognitively, and (ii) emotionally “resonating” with others' experiences. Cognitive empathy, also often called “cognitive perspective-taking,” “theory of mind,” “mentalizing,” or “mindreading,”^10^ refers to an individual's capacity to model others' experiences by making inferences about their intentions and predictions about future actions based on that mental representation. Although this cognitive process reflects one's capacity to model other people's minds, it crucially does not require emotional investment (e.g., I may understand that you are anxious but I do not feel that way myself).

Another way, then, to understand other people is to have an “insider” view by actually experiencing what the other person is experiencing. This “catching” of another person's experience is what most scholars refer to as empathy. Although there are many definitions for empathy in the psychological and philosophical literature (see Batson, 2009), most scholars broadly agree that there are two key criteria characterizing empathic responses. Firstly, empathy involves an affective capacity to recognize and resonate with others' emotions (also widely called “emotional contagion” or “affect sharing”). The affective response should be isomorphic with another person's affective state (Eisenberg and Fabes, 1990; De Vignemont and Singer, 2006). That is, one must experience the same emotion as another person, rather than simply respond emotionally to someone else's emotion (e.g., happiness in response to someone else's misfortune would not be isomorphic). This isomorphism is emphasized in the literature as distinct from related phenomena such as sympathy, which may be emotionally powerful but is usually thought of as feeling “for” rather than feeling “with.” Secondly, empathy should involve an awareness of the source of one's affective response; that is, a mechanism to distinguish between self and other. Imitation or emotional contagion alone, seen even in young infants, does not then reflect empathy (e.g., De Vignemont and Singer, 2006), as true empathy requires a more developed sense of self, agency, and other. Here, we will refer to this process as affective empathy.

Echoing the philosophical discussion above, a wide empirical research program has suggested the social and moral importance of both affective empathy and cognitive empathy, arguing that they are critical for social development and successful social interaction. Individuals with impaired (or a lack of) affective empathy are often characterized as psychopathic (e.g., Hare, 1991 as cited in Blair, 2005), and individuals with impaired theory of mind, a characteristic of autism, exhibit a host of social deficits including difficulties communicating, understanding others' thoughts and desires, recognizing and imitating others' facial expressions, among other issues (e.g., Blair, 2005). Moreover, although there might sometimes be negative consequences of increased empathy (e.g., favoring social “in-groups”; in Bloom, 2017 even goes to suggest that empathy has more costs than benefits), cognitive and affective empathic capacities in many ways provide a foundation for moral behaviors (Decety and Cowell, 2015). For instance, even short-term manipulations of cognitive perspective-taking can lead to increased feelings of social affiliation, perceived similarity, perceived closeness, intergroup understanding, desire to engage in intergroup contact, and to prosocial behaviors such as increased cooperation, sharing, comforting, and helping even in situations where prosocial attitudes might be more difficult to adopt (e.g., Stephan and Finlay, 1999; Bodenhausen et al., 2009; Wang et al., 2014)^11^.

In addition to its social importance, empathy provides an individual with knowledge about the environment without having to actually experience it oneself; for example, seeing someone get burned when they touch a hot stove or get bruised when they fall on a pavement is informative enough to attach appraisals to those situational contexts without having to experience the pain oneself (De Vignemont and Singer, 2006). This characteristic of empathy resonates with the aesthetic distance conception of fiction above, explaining how art appreciation could be a “safe space” for understanding others' difficult or taxing emotional experiences.

If art appreciation indeed enhances other-understanding, it would be reasonable to expect that we would find evidence, at least in some contexts, that engaging with art, be it viewing visual art, reading literature, or listening to music, recruits mechanisms associated with cognitive and affective empathy. For example, there may be evidence demonstrating that the neural mechanisms implicated in affective or cognitive empathy during real social interactions are also engaged when “interacting” with visual art or with fictional characters. Furthermore, art appreciation should mirror findings within the social interaction literature, such that after art-appreciation-based manipulations, we may find increases in self-reported perceived similarity and closeness, and perhaps increased degree of prosocial behavior exhibited toward an individual. Finally, we should expect that repeated “practice” or engagement with arts would develop empathy, perhaps changing aspects of one's disposition, personality, and capacity to empathize in future situations. Below, we review empirical evidence in line with each of these predictions, with the aim of demonstrating the promise and possibilities of the shift to a social practice framework in neuroaesthetics.

#### Simulation, embodiment and arts-engagement: neural mechanisms

Some researchers within neuroaesthetics have begun to reconsider arts engagement as a fully embodied, enactive experience (e.g., Freedberg and Gallese, 2007; Nadal et al., 2012), with empirical evidence suggesting the involvement of neural processes related to both perspective-taking and affective empathy during art appreciation. One such model of the role of embodied responses to visual arts is presented by Freedberg and Gallese (2007). They suggest that embodied responses occurring during art appreciation are forms of cognitive and affective simulations and, as such, play a role in facilitating an understanding of both the representational content of an artwork and of the intentions of the artist. Freedberg and Gallese provide several examples demonstrating that viewers have physical, “felt” responses to visual representations, even if those representations are abstract. For instance, the authors speculate that viewing a painting like Caravaggio's *Incredulity of Saint Thomas*, in which a man is poking at someone else's wound, or experiencing Michelangelo's *Prisoner's*, in which the figures appear “trapped” in the material out of which they are sculpted, leads to embodied responses of physical pain in the beholder. Moreover, elements within a visual artwork that simply *imply* the gestures used by the artist (e.g., canvas cuts as in artist Lucio Fontana's work, or Jackson Pollock's drip paintings) can also strongly activate the motor cortex, and are thus *felt* by beholders as actions (Battaglia et al., 2011; Umilta et al., 2012).

More evidence for action simulation during art viewing is provided by Leder et al. (2012) who demonstrate that we covertly simulate actions produced by a visual artist while we engage with the work. That is, when viewing work by Georges Seurat, for example, we may covertly “stipple” our hands, whereas while viewing art by Vincent Van Gogh, we may covertly create broader strokes with our hands. Interestingly, when the researchers experimentally manipulated participants motions to either be explicitly aligned or misaligned with painting style, preference scores were affected. That is, participants in congruent groups (stippling while viewing works in the Pointillist tradition or stroking while viewing works with strong brushstrokes) reported liking the artworks more than those in incongruent groups suggesting that incongruent motions interfered with motor resonance (Leder et al., 2012). Researchers have similarly discussed the role of embodiment with respect to music as well as the literary arts. For instance, research has demonstrated that we develop embodied understanding of characters within a literary text (for comprehensive reviews see Koopman and Hakemulder, 2015; Oatley, 2016). One such example is seen in Hsu et al. (2014) who demonstrate that immersion or “getting lost in” emotion-laden literary text—in their case, fear-inducing compared to neutral excerpts from the Harry Potter series—leads to increased activation of the medial cingulate cortex, a structure associated with affective empathy.

Together, this research suggests that engagement with visual art may prompt beholders to mentally simulate artists' actions, and to “feel” the actions and emotions depicted in a work. Although we do not mean to suggest that simulation alone implies social understanding, as is evidenced by the fact that even very young infants (or primates) imitate without a developed theory of mind (e.g., Heyes, 2001 for review) it seems to have clear social *value*. Thus, embodied responses (what some refer to as “feeling into” art) may prompt meaning-making and explicit reflection (e.g., Pelowski, 2015). Importantly however, the extent to which mirroring, simulation, and empathy affect art appreciation and even aesthetic evaluation remains understudied.

The neural processes that are implicated in embodied emotion and action simulation, namely a medial frontotemporal network involving recruitment of the bilateral anterior insula, the dorsal and middle anterior cingulate cortex (ACC), and the ventromedial prefrontal cortex (VMPFC), as well as a mirror-neuron system (MNS), are implicated in empathy and theory of mind, and are important for representing both our own and others' actions (e.g., Decety and Grèzes, 2006). For example, Wicker et al. (2003) show that overlapping areas of the ACC are activated when one is imagining, observing, and expressing a disgusted facial expression. Similarly, Morrison et al. (2004) showed overlapping activation in the anterior insula and ACC both when a person was in physical pain and when she was viewing someone else in pain^12^. These responses can be modulated by a variety of factors, including dispositional/trait empathy, relationship between empathizer and target, situational context, and emotional context (e.g., De Vignemont and Singer, 2006). For example, in one study, electromyography was used to demonstrate that people with high affective trait empathy were more likely to automatically imitate happy and angry pictures of faces during passive viewing than people with low affective trait empathy (Rymarczyk et al., 2016).

With respect to visual art, a recent study similarly showed that trait empathy correlated to both physiological (facial electromyography and skin conductance responses) and behavioral responses to art (valence, preference, interest) (Gernot et al., 2017). Specifically, they showed that individuals who are high in emotion contagion are more moved by, interested in, and enjoy visual art. These high emotion contagion individuals also reacted more strongly to emotion congruent aspects of the visual art (e.g., they smiled while engaging with positive valence work and frowned when engaging with negative valence works). Similar findings have been reported within music, in which individual differences in empathetic capacities relate to understanding and interpretation of emotional expressivity and intentionality in music (Wöllner, 2012; Baltes and Miu, 2014). In this way, the empirical evidence points to a role for empathy in synchronizing emotion-relevant perceptions and actions among individuals, perhaps for understanding others more effectively, a skill art engagement may facilitate.

Another important set of neural structures—specifically within a lateral frontotempoparietal network (relevant regions include: lateral and medial PFC, lateral and medial parietal cortex, and medial temporal lobe, temporoparietal junction, and posterior superior temporal sulcus)—have been shown to correlate with tasks related to cognitive empathy such as action observation, imitation, self-recognition, impersonal moral and social reasoning, reappraisal by focusing on physical events, and categorizing affect in facial expressions (e.g., Lieberman, 2007). There is also a connection between this network and the mirror neuron network discovered in primates. In primates, mirror neurons activate both when the primate performs a goal-directed action and when it observes the experimenter performing the same action (Gallese et al., 1996). In humans, homologous regions of cortex (premotor cortex, LPFC, LPAC, DMPFC) similarly respond both to action observation and to imitation (e.g., Carr et al., 2003). Along with the regions that are implicated in embodied emotion and action simulation described above, these structures may be target regions of interest for neuroaesthetics.

The evidence linking neural processes recruited during other-understanding to art appreciation as reviewed above is promising. Perhaps the mirror neuron system (and other neural processes related to mentalizing as reviewed above) play an important role in enabling an experiential understanding of the content of a visual artwork as well as some of the artist's intentions (Freedberg and Gallese, 2007). Though more research is crucial, the findings up to this point suggest that engaging with art involves processes relevant to the attribution of mental states to others (Steinbeis and Koelsch, 2009; Koopman and Hakemulder, 2015), and this suggests that art appreciation is deeply connected to other-understanding.

#### (Pro)social effects of art appreciation

Based on the presented evidence, if cognitive and affective empathic processes are recruited during art appreciation, just as is observed for empathy manipulations, we should observe increases in measures such as self-reported perceived similarity, closeness, or degree of prosocial behavior exhibited toward an individual after arts-appreciation-based manipulations. Again, the literary arts are an example domain where research has been particularly comprehensive. The effect of reading literature, and more specifically, narrative fiction on empathy and other-understanding has recently received widespread attention (see Koopman and Hakemulder, 2015; Oatley, 2016 for comprehensive reviews). For example, Kotovych et al. (2011), find that the “challengingness” of the text, operationalized as the complexity of characters and number of ambiguities in a text, helps readers better identify with, feel more connected to, and understand a character more deeply. One explanation for such an effect is that when a literary text leaves more information about the narrator's mental life implicit and ambiguous, readers may be more likely to draw from their own experiences, resulting in a seemingly stronger connection with and understanding for an individual.

Further, psychologists have demonstrated both correlational and causal effects of reading narrative on various measures of empathy. Measures of empathy in these cases include the “Reading the Mind in the Eyes Test,” which probes one's ability to discern another individual's thoughts from their eyes alone (RMET; Baron-Cohen et al., 2001), or the Yoni test, which asks participants to identify others' affective and cognitive states from facial expressions (Shamay-Tsoory and Aharon-Peretz, 2007). Researchers have demonstrated that individuals who spend more time reading literary or narrative fiction compared to non-fiction tend to score higher on such tests suggesting that extended “practice” reading narrative fiction may cultivate one's capacity for understanding others (e.g., Mar et al., 2006; Panero et al., 2016). And, a recent series of experiments by Kidd and Castano (2013) demonstrated that individuals who were tasked with reading a “literary” short story that is characterized by unconventional syntax, ambiguity, and semantic features scored higher on the RMET and Yoni tasks after the reading exercise compared to those who read a popular fiction or nonfiction short story. This finding demonstrates that even brief exposure to the arts might promote other-understanding.

Importantly, empathy-related processing during arts appreciation across domains (e.g., beyond just the literary arts) also seems to lead to increased prosocial behavior. For example, Sze et al. (2012) demonstrated that after watching film clips that induced empathetic concern, individuals tended to be more charitable. Interestingly, these prosocial effects were partially mediated by age such that older participants were more charitable than their younger counterparts. Although not directly related to film appreciation *per se* (as film in this case was merely a stimulus meant to elicit empathetic concern), it is suggestive both of the power of film and the cultivation of prosocial tendencies with art experience. Film's power to move the viewer in this way has also been associated with increased feelings of intergroup connectedness and understanding (Oliver et al., 2015). Likewise, some research suggests that chills induced by music lead to more altruistic behavior, though more research is needed to tease apart the influence of factors like mood (Fukui and Toyoshima, 2014). Taken together, these findings suggest the importance of a continuing research program on the (pro)social implications of arts engagement.

Although these effects seem promising, many of the claims about empathy cultivated through art appreciation are contested. For instance, some researchers have been unable to replicate the causal effects (most recently, Panero et al., 2016), noting, like Bullot and Reber (2013), that a brief encounter is typically “shallow” and is unlikely to have significant impacts on cognitive or affective empathy. This is not altogether surprising as measures like the RMET are likely relatively stable across time. And, even if it appears that art engagement increases state empathy—that is, empathic responses during the interaction—the single engagement may not cultivate empathy in the long term in real-life scenarios the way that researchers hope. It is not inconceivable that an individual connects to fictional characters described as in a particular situation, but would not connect to real people in that same situation^13^. Furthermore, it is theoretically unclear why individuals who read a story just once, or even those who are well-read, should be better attuned to discriminating facial expressivity *per se*. Rather, it might be that narrative fiction develops imaginative capacity. In fact, research by Johnson (2012) finds that reading fiction can actually lead to decreased perceptual accuracy in discriminating fearful emotions. Johnson speculates that such reduced discriminability is likely due to a bias in attributing emotions, particularly ones congruent with a prosocial behavior, to ambiguous expressions. Similarly, research attempting to quantify the effects of both brief and longer-term art encounters on empathy and patient outcomes for medical professionals is contested and still underdeveloped (e.g., Perry et al., 2011; Yang and Yang, 2013; Kelm et al., 2014). Finally, there is conflicting evidence on the extent to which thrills-like responses affect schemas and behavior. For instance, the physical chills response that some individuals report in response to music as well as to visual art and literature does not always seem to differentially affect prosocial behaviors or self concept, relative to artworks that do not elicit chills (Konecni et al., 2007). Thus, more empirical studies are needed to systematically address how art appreciation actually affects other-understanding.

#### Summary

We began this section by reviewing philosophical views that hold or imply that art appreciation is socio-epistemically valuable insofar as it cultivates other-understanding through processes like emotional sharing or imaginative understanding. Following these ideas, psychologists and neuroscientists have begun to empirically assess whether and how art appreciation deepens other-understanding. Empirical research has up to this point demonstrated that art appreciation engages similar psychological processes that are involved in social interaction, such as emotional resonance, mental state attribution, and cognitive perspective taking. Furthermore, we reviewed evidence that showed that increased “practice” appreciating the arts, arts-appreciation “interventions” (as in medical school curricula), and even “basic exposure” to the arts (as in Kidd and Castano, 2013) increased individual's capacities for other-understanding. Although it is promising, the empirical and philosophical research centered on the relationship between art appreciation and other-understanding is still limited in its scope, quantity, and specificity. Particularly important will be to develop robust (perhaps more longitudinal) methodologies that demonstrate the processes by which arts appreciation cultivates other-understanding as well as its relationship to self-understanding, leading a flourishing life, and other socio-epistemic skills.

## Looking ahead

In this paper, we aimed to highlight how understanding the power of the arts in our lives requires going beyond the current aesthetics-focused conception of the outcomes of art appreciation. Rather than neuroaesthetics models which focus nearly exclusively on judgments of beauty, preference, or liking as the primary outcomes of art appreciation, we should set ourselves to better understanding the range of socio-epistemic outcomes of such engagement. Here, we have focused on self-understanding and other-understanding as such outcomes, but do not intend to limit the potential of this framework shift to just these outcomes. Rather, we aimed to provide evidence for the fruitfulness of neuroaesthetics adopting a more comprehensive approach to the outcomes of art appreciation that mirror the richer conceptions of art engagement found in philosophy, art history, and art criticism, which understand art as an embodied, enactive, social practice.

Importantly, such an approach does not discount prior empirical research, but refocuses its aim around socio-epistemic skills developed within arts practices. In thinking of the arts as social practices that people engage in, we can come to better understand how they serve a variety of social and cultural values. We hope this approach inspires empirical research to more fully investigate the specific ways in which the processes underlying art engagement cultivate socio-epistemically valuable skills. That is, how do specific emotional experiences lead to self-understanding? To other-understanding? And to other socio-epistemic values? How does engagement with different art forms relate to distinct socio-epistemic values? Does engagement with literary art, for example, more promote a particular set of values, compared to practiced engagement with the visual arts or music?

To answer these questions, researchers will need to go beyond the typical unitary measures of preference after a single exposure, and instead employ more longitudinal designs incorporating both state and trait based measures. Take for example a researcher interested in whether and how engaging with particular form of visual art (e.g., art depicting minority groups such as American Indians) may deepen ones cultural understanding and appreciation. To go beyond standard designs, one might consider (a) encouraging viewers to engage with each artwork for longer periods of time (e.g., at least 1 minute), (b) comparing lab findings to naturalistic settings (e.g., conducting experiments in both settings to determine generalizability of lab results) and (c) combining methodologies (e.g., eye tracking, physiology, EEG, subjective self-reports such as being moved, interest, emotional state, and written reflections). Possible individual difference measures that researchers may employ include tests that measure capacity for cognitive and affective empathy [e.g., the Empathy Quotient (EQ; Lawrence et al., 2004), the Interpersonal Reactivity index (IRI; Davis, 1980), or the questionnaire of affective and cognitive empathy (QCAE, Reniers et al., 2011)], tests that measure state and dispositional aspects of self-awareness [e.g., the Mindfulness Attention Awareness Scale (MAAS; Brown and Ryan, 2003), self concept clarity questionnaires, tolerance for uncertainty, Webster and Kruglanski, 1994], tests that measure emotion perception and regulation (e.g., the scale of subjective emotion experience (See; as in Pelowski et al., 2017), and subjective self reports relevant to one's art experience including art expertise, interest, reflections and insights. Furthermore, researchers may adopt experimental techniques from the mindfulness and meditation literature, which similarly aims to demonstrate the perceptual, cognitive, and emotional effects of mindfulness practices as compared simply to mindful states. Thus, we see our reframing as an exciting opportunity for researchers to be creative in designs (see Table 2 for examples of open questions).

**Table 2:** Open questions.

**How does art appreciation promote *self*-understanding?**	**How does art appreciation promote *other*-understanding?**
(How) Are the processes relevant to self-understanding (e.g., self-reflection, self-awareness, metacognition, self-concept/schema/belief revision, insight, epiphany) recruited during art appreciation? Do individuals with more art expertise possess stronger self-reflective skills? What brain regions and networks are involved in self-understanding as it relates to art appreciation? A candidate network to investigate is the default mode network (e.g., as reported in Vessel et al., 2012, 2013), and cortical midline structures (e.g., DMPFC, OMPFC, anterior and posterior cingulate cortex, as in Northoff and Bermpohl, 2004; Northoff et al., 2006). How do behavioral and physiological outcomes of art appreciation (e.g., being moved, tears, chills, thrills, arousal) indicate self-referential processing and self-understanding? Under what circumstances do processes like self-reflection occur during art appreciation? For example, how do current states, traits, and art content (e.g., style, features, representation) interact to facilitate self-understanding? Are these interactions art-domain specific or general? How might mindset manipulations (e.g., self or other directed focus) during art-appreciation increase self-reflection and understanding? How do other socio-epistemic skills cultivated by art appreciation (see Table 1 for examples) interact with self-understanding? How can cognitive neuroscience and psychology inform art (appreciation) therapies that focus on cultivating self-understanding? How does art creation (or exercising creativity through the arts) relate to the cultivation of self-understanding? Are the processes similar to art appreciation?	(How) Are the processes relevant to other-understanding (e.g., perspective-taking/cognitive empathy, imitation/mimicry, affective empathy/emotional resonance) recruited during art appreciation? Do individuals with more art expertise possess stronger empathetic tendencies? What brain regions and networks are involved in other-understanding as it relates to art appreciation? Candidate systems include the medial frontotemporal network (e.g., anterior insula, dorsal and middle anterior cingulate cortex, VMPFC, human MNS) as well as the lateral frontotempoparietal network (e.g., lateral and medial PFC, lateral and medial parietal cortex, medial temporal lobe, temporoparietal junction, and posterior superior temporal sulcus). What are behavioral and physiological indicators of other-understanding? Examples include emotional resonance (e.g., emotion-congruent expressions as measured by fEMG in Pelowski et al., 2017), and covert or overt mimicry. How are behavioral and physiological outcomes of art appreciation (e.g., being moved, tears, chills, thrills, arousal) prompted by other-understanding? Menninghaus et al. (2015) suggest films with prosocial elements lead viewers to be moved. How might this generalize to other art-domains? Research shows perspective-taking manipulations lead to increased intergroup understanding and affiliation. How might such manipulations during art-appreciation increase other-understanding? How do other socio-epistemic skills cultivated by art appreciation (see Table 1 for examples) interact with other-understanding? How can cognitive neuroscience and psychology inform art (appreciation) therapies that focus on cultivating other-understanding?

*Outstanding questions for investigating the psychological and neurobiological relationships between self-understanding, other-understanding, and art appreciation*.

Further, this kind of “art as social practice” approach encourages scientists to view art engagement, generally, be it appreciating or creating, as a form of knowledge acquisition and production. Although we focused here on art appreciation, we believe our approach generalizes to art creation. Like art appreciation, art making involves practices which integrate embodied and “mental” activities so as to render the two inseparable. In fact, the philosophical and psychological research on creation and creativity recognizes and investigates such processes of creative practice associated with individual development more so than does the research on art appreciation.

Finally, we believe that focusing on the socio-epistemic skills cultivated through art engagement highlights the important role art plays in our lives, and the need to advocate for arts education programs. Through this kind of research program, we should come to better understand the arts as socially valuable. We suggest that empirical research can be used to show that engagement with art has social and personal value, rather than monetary or economic value, the cultivation of which is important to us as individuals, and as communities.

## Author contributions

All authors listed have made substantial, direct, intellectual contributions to the work, and approved it for publication.

### Conflict of interest statement

The authors declare that the research was conducted in the absence of any commercial or financial relationships that could be construed as a potential conflict of interest.

## Footnotes

^1^Recent arguments by influential researchers such as Pearce et al. (2016) suggest that neuroaesthetics is often concerned not with explaining art appreciation, but rather with understanding the aesthetic qualities of objects that include the arts. However, findings within the aesthetic sciences are often used to explain *art* appreciation, specifically (e.g., Pelowski et al., 2016 published a review article titled “Visualizing the Impact of Art: An Update and Comparison of Current Psychological Models of Art Experience” in which they do just that).

^2^While it may be that the kinds of social practices we are talking about relate to “artworld” institutions, practices are logically independent of and prior to institutions (see MacIntyre, 1999 for the relationship between practices and institutions).

^3^The kind of theoretical shift we recommend—toward understanding the arts as practices—is also related to Noë's (2015). There, he develops an account of the arts as *organized activities*, insofar as they are: (1) natural or primitive, (2) “arenas for the exercise of attention, looking, listening, doing, undergoing” (p. 6), (3) structured and organized in time, (4) emergent, and which (5) have a function and (6) are a source of pleasure for those who engage in them (pp. 4–5). This approach is similar to the social practice account in that it is interested in the role of the arts in structuring a well-functioning or flourishing human life. It differs on the strength of the emphasis placed on the embodied nature of the arts, and in the expressed biological and “natural” interpretation it gives to these practices through the notion of “organizing” that it employs.

^4^See Stolnitz (1992) for discussion of the philosophical debate about aesthetic cognitivism, which is concerned with whether we can learn from or know through art appreciation.

^5^In doing so we do not claim that these are the *only* valuable socio-epistemic skills developed by the social practices of the arts or arts appreciation. For example, the “Seven C's” identified by Koelsch (2014) (social contact, social cognition, co-pathy, communication, coordination of actions, cooperation, social cohesion) is a taxonomy of what the author refers to as social functions of music. Similarly, other researchers including Panksepp (2009) highlight the social importance of music evolutionarily, particularly in its capacity to evoke social emotions.

^6^Another hypothesis about this focus on moral knowledge may come from the overlap in moral and hedonic processing, evidence for which may be found in Tsukiura and Cabeza (2010).

^7^Kieran's argument draws on the rich discussion of moral understanding and art appreciation, especially that of Iris Murdoch and Martha Nussbaum. Iris Murdoch argued that engagement with and creation of art (especially painting and literature) hone moral *perception* by tuning the perceiver to the salient features of moral reality; the arts make one's moral perception more discriminating and discerning. That is to say, engagement with the arts develops one's ability to see the world as it truly is, making art “the most educational of all human activities.” (1970) In *Love's Knowledge* Nussbaum contends that moral imagination, necessary to good moral judgment (and seeing the world as it truly is), is similar to artistic imagination (1990). She explicitly links the type of fine-grained attention to detail and ability to “see” the world in morally complex and nuanced ways cultivated by arts appreciation with the development of self and other-understanding.

^8^There is some disagreement among philosophers about what cognitive process best characterizes this emotional-engagement, theorists variably refer to identification, empathy, sympathy, and mental simulation (see Giovannelli, 2005).

^9^Some researchers have gone so far as to speculate on the socio-cultural benefits of arts engagement in relation to other-understanding. In his book, *The Better Angels of our Nature*, Pinker (2012) speculates that a decrease in contemporary violence can be partially attributed to increased literary consumption, relying on the notion that perspective-taking is fundamental to reading literature and that it leads to increased empathy and other-understanding.

^10^We gloss over here some of the nuances that distinguish each of these terms. For instance, theory of mind is most often discussed in a developmental context, in contrast to cognitive perspective-taking and cognitive empathy. However, for the most part, they refer to the same/a very similar process.

^11^Heyes (2001) provides an analysis of theories and evidence describing the relationship between imitation, theory of mind, and social cognition. Heyes points out “although it is plausible that the experience of imitating and being imitated contributes to the development of theory of mind, there is not currently a well-supported theory specifying the nature of the contribution” (p. 260).

^12^Additionally, Singer et al. (2006) demonstrated that the proposed neural networks subserving empathy indeed represent “true” empathizing with another person, rather than just imagining one's own emotional experience. They first engaged participants in a game in which confederates played either fairly or unfairly. They then showed the same participants videos of their fair and unfair partners experiencing pain, while simultaneously measuring participants neural activity. Interestingly, all participants empathized with fair players, but only female participants empathized with the pain felt by unfair players experienced. In contrast, males seemed to experience more joy (evidenced by activation of reward circuitry), indicating their seeming desire for revenge against unfair players.

^13^Philip Sidney wrote a sonnet about just this point in the 1580s: http://www.bartleby.com/358/46.html
